# Hormonal contraceptives and cancer risk in women with a *BRCA1* or *BRCA2* pathogenic variant

**DOI:** 10.1007/s12282-026-01890-z

**Published:** 2026-08-18

**Authors:** Chen Nahshon, Leen Fahoum, Dana Hadar, Hila Cohen, Rachel Michaelson-Cohen, Shira Regev, Meirav Schmidt, Wisam Assaf, Steven A. Narod, Yakir Segev

**Affiliations:** 1 The Ruth and Bruce Rappaport Faculty of Medicine, Technion Israel Institute of Technology, Haifa, Israel; 2 Research Authority, Clalit Health Care Organization, Carmel Medical Center, Haifa, Israel; 3 Medical Genetics Institute, Department of and Gynecology, Shaare Zedek Medical Center, Faculty of Medicine, Hebrew University of Jerusalem, Jerusalem, Israel; 4 Dalla Lana School of Public Health, University of Toronto, Toronto, ON Canada; 5 Women’s College Research Institute, Women’s College Hospital, Toronto, ON Canada; 6 Division for Gynecological Oncology, Department of Obstetrics & Gynecology, Carmel Medical Center, Haifa, Israel

**Keywords:** BRCA, Ovarian cancer, Breast cancer, Contraceptives, Pills, IUD

## Abstract

**Background:**

Women with *BRCA1/2* pathogenic variants face increased risks of breast and ovarian cancers. Our study aimed to evaluate the impact of hormonal contraceptive use on these risks.

**Methods:**

This matched cohort study included 1677 women who were found to carry a *BRCA1*/*2* pathogenic variant. A Cox proportional hazards model was used to estimate hazard ratios associated with oral contraceptive use and with levonorgestrel-releasing intrauterine device use.

**Results:**

Among *BRCA1* carriers (n = 990), oral contraceptive use was not associated with breast cancer risk (HR = 0.90, 95% CI 0.77–1.05, p = 0.2). Among *BRCA2* carriers (n = 687), oral contraceptive use was associated with reduced breast cancer risk (HR = 0.80, 95% CI 0.65–0.99, p = 0.04), although duration-stratified analyses did not demonstrate a consistent dose–response relationship. Oral contraceptive use was associated with a decrease in the risk of ovarian cancer in *BRCA2* carriers (HR = 0.57, 95% CI 0.35–0.94, p = 0.028), with the strongest protective effect observed among *BRCA2* carriers who used an oral contraceptive for more than 5 years (HR = 0.13; 95% CI 0.02–0.92). The protective effect of oral contraceptive use was less profound for *BRCA1* carriers. The use of a levonorgestrel-releasing intrauterine device was not associated with breast or ovarian cancer risk.

**Conclusion:**

The use of an oral contraceptive was associated with reduced risks of breast and ovarian cancer among BRCA2 carriers, although the observed association with breast cancer should be interpreted cautiously given the absence of a consistent duration-response pattern. There was no increase in the risk of breast cancer among *BRCA1* carriers who used an oral contraceptive. No association with either cancer was found among users of a levonorgestrel-releasing intrauterine device.

**Supplementary Information:**

The online version contains supplementary material available at https://doi.org/10.1007/s12282-026-01890-z.

## Introduction

Germline pathogenic variants in *BRCA1* and *BRCA2* are associated with elevated lifetime risks of breast cancer (approximately 70% for both genes) and for ovarian cancer (44% and 17% respectively) [1, 2]. Mutations in these genes are increased in frequency in the Ashkenazi Jewish population, due to the high prevalence of three founder mutations (two in *BRCA1* and one in *BRCA2*). The *BRCA2* pathogenic variant 6174delG is associated with a lower risk of breast cancer in comparison with other *BRCA2* pathogenic variants [3].

Since January 2020, a population screening test for founder *BRCA1* and *BRCA2* pathogenic variants is available in Israel and is subsidized for women with partial or full Ashkenazi ethnicity, aged 25 years and above, without considering family history [4]. The test panel includes 14 common pathogenic variants in *BRCA1* and *BRCA2* in the Israeli population [4].

Given the high case-fatality of ovarian cancer and the lack of effective screening protocols, primary prevention is strongly recommended for women who test positive. Specifically, risk-reducing bilateral salpingo-oophorectomy is advised between the ages of 35 and 40 for women with a *BRCA1* pathogenic variant and between ages 40 and 45 for women with a *BRCA2* pathogenic variant [5]. Although bilateral salpingo-oophorectomy is an effective preventive strategy, some women prefer to wait until the age of natural menopause to avoid the symptoms of surgical menopause and/or to maintain fertility.

In general, testing for *BRCA1* and *BRCA2* is not done until after age 18 and typically after age 25. However, some young women wish to know if taking an oral contraceptive is associated with an increased risk of hereditary breast cancer, and if so, whether they should avoid taking an oral contraceptive if they carry a pathogenic variant. For some, this information is a motivation for testing before age 25. This is of particular importance to women with a pathogenic variant who plan to undergo risk reduction salpingo-oophorectomy, but who wish to retain their breasts.

Alternative strategies that could potentially delay the need for oophorectomy (and premature menopause), while reducing cancer risk, would be valuable. Some researchers have suggested bilateral salpingectomy as an alternative to salpingo-oophorectomy [6]. Another possible strategy is the use of oral contraceptive pills, which is an established protective factor against ovarian cancer, both among high-risk women and among the general population [7–11].

In recent decades, the use of intrauterine devices has increased substantially [12]. The rising popularity of intrauterine devices is attributed to their high efficacy, user satisfaction, reversibility, and ease of use [13]. Two intrauterine devices formulations are commercially available in Israel: the levonorgestrel-releasing intrauterine device and the copper intrauterine device. The levonorgestrel-releasing intrauterine device releases progestin, leading to endometrial suppression and cervical mucus thickening, whereas the copper intrauterine device induces a local inflammatory response to prevent fertilization [12]. The most commonly used hormonal intrauterine device is the five-year, 52 mg levonorgestrel bearing device [14]. Studies investigating the association between intrauterine device use and ovarian cancer risk have shown inconsistent results [15–19]. To date, data regarding the association between intrauterine device use and ovarian and breast cancer risk among *BRCA1/2* carriers is scarce. A single study evaluating long-acting contraceptive methods in this population found no significant association between intrauterine device use and the risk of ovarian cancer [20]. This report did not study the effect of hormonal intrauterine devices on breast cancer risk.

Carriers of *BRCA1/2* pathogenic variants face complex decisions regarding contraceptive options. Combined oral contraceptives have been demonstrated to significantly reduce the risk of ovarian cancer; however, their daily use may pose challenges related to adherence and potential side effects. In a recent study, long term oral contraceptive use was associated with a modest increase in the risk of breast cancer in *BRCA1* carriers, but not *BRCA2* carriers [21]. In contrast, the levonorgestrel-releasing intrauterine device offers several advantages, yet its protective effect against ovarian cancer has not been established. Furthermore, the impact of levonorgestrel-releasing intrauterine device on breast cancer risk among *BRCA1/2* carriers remains uncertain.

The primary aim of the present historical cohort study was to investigate the relationship between oral contraceptives and levonorgestrel-releasing intrauterine device use and the risks of ovarian and breast cancer in *BRCA1* and *BRCA2* pathogenic variant carriers. Clarifying these associations could help guide contraceptive counselling and risk-reduction strategies in this high-risk population.

## Material and methods

### Data collection

The subjects in this historical cohort study were members of the Clalit Health Services plan in Israel. Clalit is the largest healthcare provider in Israel, with an estimated 5,400,000 members, representing 50% of the Israeli population, as previously described in a nationwide Clalit-based study [22]. Approval for the study was obtained from the Institutional Review Board of Carmel Medical Center (Protocol No. 0147–24-CMC).

The study used de-identified data from Clalit members identified to carry a *BRCA1* or *BRCA2* pathogenic variants, aged 18 and above, between January 2000 and December 31, 2024.

Genetic testing was performed in the following cases: 1) Population survey: any woman with a partial or full Ashkenazi origin, aged 25 years and above, with no self-reported family history; 2) Subsequent to genetic counseling due to a family cancer history. 3) A personal history of breast or ovarian cancer.

The data were securely stored and processed within Clalit Health Services protected virtual environment.

The collected data included various demographic and clinical variables, including date of birth, body mass index (BMI), smoking status, number of births and the use of hormonal replacement therapy. Data were based on ICD-10 diagnoses and included dispensation of medications. The data on the use of oral contraceptives included the date of first use and the date of last use, but not the specific formulation. Duration of use was taken as the time elapsed from first use to last use. Additional data included patients who used levonorgestrel-releasing intrauterine device during this time period. The data on levonorgestrel-releasing intrauterine device users including demographics, genetic pathogenic variant type, age at first levonorgestrel-releasing intrauterine device use.

To reduce the possible influence of a significant time trend associated with oral contraceptive use in Israel, we conducted a matched analysis. Each woman who used an oral contraceptive was age-matched to one woman who did not use an oral contraceptive, using nearest‐neighbor matching without replacement. Matching was performed on birth year (± 1 year). The matched pairs were the subjects of the primary analysis. The matched cohort was then analyzed using a time-dependent Cox proportional hazards model. Oral contraceptive use and levonorgestrel-releasing intrauterine device use were treated as time-dependent covariates. To evaluate the impact of duration of oral contraceptive use on cancer risk, participants were categorized into three groups: short-term users (≤ 2 years), medium-term users (2–5 years), and long-term users (≥ 5 years).

Descriptive data including breast and ovarian cancer diagnosis following contraceptive use were extracted and analyzed.

### Statistical analysis

Baseline characteristics were compared between the groups by one-way ANOVA or Chi-squared, where applicable. The Cox proportional hazards model was used to assess the association between oral contraceptive use and the incidence of breast and ovarian cancer. Each analysis was performed separately for *BRCA1* and *BRCA2* pathogenic variants carriers, and separately for breast and ovarian cancer.

For the breast cancer analysis, follow-up began at age 18 and continued until the first of the following: occurrence of breast cancer, bilateral mastectomy, death or end of study period (December 31, 2024).

For the ovarian cancer analysis, follow-up began at age 18 and continued until the first of the following: occurrence of ovarian cancer, bilateral salpingo-oophorectomy, death or end of study period.

Contraceptive exposure was modeled as a time-dependent covariate. Women who used oral contraceptives or levonorgestrel-releasing intrauterine devices prior to age 18 were considered exposed from baseline. Those who did not use hormonal contraception prior to age 18 were considered unexposed (non-user) from age 18 until completion of follow-up or initiation of hormonal contraceptive use. From the time of first use onwards, women were classified according to their contraceptive method. Hormone replacement therapy exposure was included as a fixed (time-independent) covariate (ever vs. never use), due to limited data regarding the precise timing of initiation and discontinuation. All hazard ratios were adjusted for hormone replacement therapy use.

Results are reported as hazard ratios (HRs**)** with 95% confidence intervals (CIs).

For each cancer type we plotted separate Kaplan–Meier curves stratified by contraceptive exposure group and gene status to visualize differences in cumulative cancer incidence over time. All analyses were conducted using R statistical software**.**

Given that the analyses were based on a priori hypotheses regarding breast and ovarian cancer risk in BRCA1/2 pathogenic variant carriers, no formal adjustment for multiple comparisons was applied. However, findings with borderline statistical significance should be interpreted cautiously, with emphasis placed on effect sizes and confidence intervals rather than p-values alone.

In accordance with the journal’s guidelines, we will provide our data for independent analysis by a selected team by the Editorial Team for the purposes of additional data analysis or for the reproducibility of this study in other centers if such is requested.

## Results

A total of 1677 women were included in the matched analysis, including 839 oral contraceptive users and 838 non-users. Baseline characteristics are shown in Table 1. Smoking status, BMI, parity, and year of genetic diagnosis did not differ significantly between the two groups. Women who used an oral contraceptive were more likely to have used hormone replacement therapy than contraceptive non-users (25% vs. 14%, p < 0.001). 511 of the women used a levonorgestrel-releasing intrauterine device. Characteristics of women who did and did not use a levonorgestrel-releasing intrauterine device are summarized in Supplementary Table S1. The median age at first use of a levonorgestrel-releasing intrauterine device use was 37 years (IQR 32–41 years).

**Table 1:** Characteristics of women according to OCP Use (matched)

Characteristic	OCP users N = 839	No OCP users N = 838	p-value
Pathogenic variant type, n (%)			0.012
BRCA1	470 (56%)	520 (62%)	
BRCA2	369 (44%)	318 (38%)	
Smoking status, n (%)			0.5
Non-smoker	653 (78%)	654 (78%)	
Past smoker	114 (14%)	102 (12%)	
Active smoker	72 (8.6%)	82 (9.8%)	
OCP subgroups			–
OCP up to 2 years (n,%)	161 (19%)	–	
OCP 2–5 years (n,%)	399 (48%)	–	
OCP 5 years and above (n,%)	279 (33%)	–	
OCP duration, median (IQR), y	4.45 (0.76,9.43)	–	–
Age at first OCP use, median (IQR)	29 (22,39)	–	–
Year of birth, median (IQR)	1974 (1,966,1,985)	1973 (1,965,1,984)	0.2
BMI, mean (SD)	26.02 (5.62)	26.10 (5.48)	0.5
Parity, mean (SD)	3.16 (1.62)	3.22 (1.62)	0.7
HRT use, n (%)	210 (25%)	117 (14%)	<0.001
Age at BRCA diagnosis, median (range)	44 (18,74)	44 (18,76)	0.2
Year at BRCA diagnosis, median (range)	2,020 (1,999,2,024)	2,020 (1,999,2,024)	0.3
Ovarian cancer diagnosis, n (%)	36 (4.3%)	52 (6.2%)	0.08
Age at ovarian cancer, median (range)	50 (24,73)	51 (23,77)	0.9
Year at ovarian cancer diagnosis (range)	2,016 (1,975, 2,024)	2,018 (1,987, 2,024)	0.1
Breast cancer diagnosis, n (%)	237 (28.2%)	288 (34.4%)	0.007
Age at breast cancer, median (range)	43 (19,70)	42 (19,71)	0.2
Year at breast cancer diagnosis (range)	2015 (1975,2024)	2015 (1975,2024)	0.7
Bilateral salpingo-oophorectomy, n (%)	284 (34%)	269 (32%)	0.4
Age at BSO, median (IQR)	46.5 (40,53)	47 (42,55)	0.2
Bilateral mastectomy, n (%)	92 (11%)	81 (9.7%)	0.4
Age at mastectomy, median (IQR)	41.5 (37,49)	41 (36,50)	0.8

**Fig. 1:**
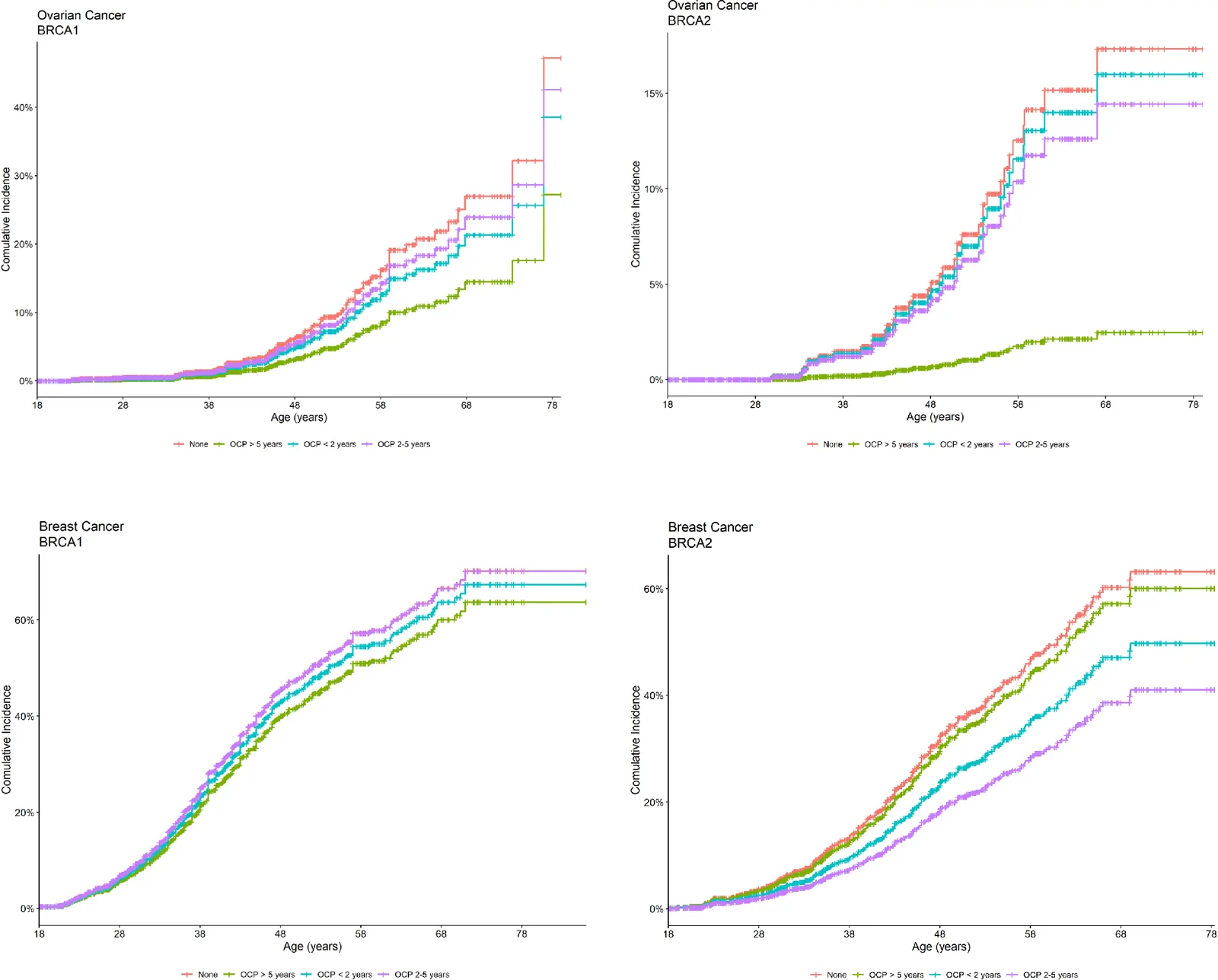
Kaplan–Meier curves stratified by oral contraceptive use and BRCA pathogenic variant type. OCP, oral contraceptives. **A** Ovarian cancer among BRCA1 pathogenic variant carriers. **B** Ovarian cancer among BRCA2 pathogenic variant carriers. **C** Breast cancer among BRCA1 pathogenic variant carriers; the non-oral contraceptive group curve overlaps with the oral contraceptive exposure group of 2–5 years. **D** Breast cancer among BRCA2 pathogenic variant carrierst

Multivariable Cox regression analyses evaluating cancer risk according to contraceptive exposure are presented in Tables 2, 3, 4 and 5. Figure 1 presents the Kaplan–Meier curves stratified by cancer diagnosis, oral contraceptive group and *BRCA* pathogenic variant type.

**Table 2:** Risk factors for breast cancer according to contraceptives, BRCA1 PV variants, n = 990

Characteristic		Breast cancer		
		HR^*1*^	95% CI^*1*^	p-value
Nothing		–	–	ref
OCP overall		0.90	0.77, 1.05	0.2
OCP subgroups	OCP up to 2 years	0.93	0.70, 1.23	0.6
	OCP 2–5 years	1.00	0.67, 1.48	> 0.9
	OCP 5 years and above	0.85	0.67, 1.07	0.2
HRT use		0.92	0.69, 1.22	0.5

**Table 3:** Risk factors for ovarian cancer according to contraceptives, BRCA1 PV variants, n = 990

Characteristic		Ovarian cancer		
		HR^*1*^	95% CI^*1*^	p-value
Nothing		–	–	ref
OCP		0.70	0.48, 1.03	0.071
OCP subgroups	OCP up to 2 years	0.78	0.47, 1.29	0.3
	OCP 2–5 years	0.87	0.34, 2.24	0.8
	OCP 5 years and above	0.51	0.21, 1.23	0.14
HRT use		0.80	0.37, 1.73	0.6

**Table 4:** Risk factors for Breast Cancer according to contraceptives, BRCA2 PV variants, n = 687

Characteristic		Breast cancer		
		HR^*1*^	95% CI^*1*^	p-value
Nothing		–	–	ref
OCP		0.80	0.65, 0.99	0.040
OCP subgroups	OCP up to 2 years	0.72	0.48, 1.09	0.12
	OCP 2–5 years	0.55	0.30, 1.02	0.059
	OCP 5 years and above	0.98	0.75, 1.28	0.9
HRT use		0.47	0.28, 0.79	0.005

**Table 5:** Risk factors for ovarian cancer according to contraceptives, BRCA2PV variants, n = 687

Characteristic		Ovarian cancer		
		HR^*1*^	95% CI^*1*^	p-value
Nothing		–	–	ref
OCP		0.57	0.35, 0.94	0.028
OCP subgroups	OCP up to 2 years	0.92	0.48, 1.77	0.8
	OCP 2–5 years	0.83	0.25, 2.74	0.8
	OCP 5 years and above	0.13	0.02, 0.92	0.041
HRT use		0.93	0.31, 2.79	0.9

Among *BRCA1* carriers (n = 990), oral contraceptive use was not associated with breast cancer risk (HR for ever/never use = 0.90, 95% CI 0.77–1.05, p = 0.2). When stratified by duration of oral contraceptives use, neither short-term use (≤ 2 years), medium-term use (2–5 years) or long-term use (≥ 5 years) showed a significant association with breast cancer risk. Among *BRCA2* carriers (n = 687) any oral contraceptive use was associated with a small but statistically significant reduction in breast cancer risk (HR 0.80, 95% CI 0.65–0.99, p = 0.040). In subgroup analyses, short (≤ 2 years), medium (2–5 years) and long-term oral contraceptive use (≥ 5 years) showed no significant effect on breast cancer risk (HR 0.72, p = 0.12, HR 0.55, p = 0.06 and HR 0.98, p = 0.9, respectively).

In *BRCA1* carriers, ever use of oral contraceptives was not significantly associated with ovarian cancer risk (HR 0.70, 95% CI 0.48–1.03, p = 0.071), For ovarian cancer in *BRCA2* carriers, ever-use of an oral contraceptive was associated with a significantly reduced risk of ovarian cancer (HR 0.57, 95% CI 0.35–0.94, p = 0.028). A strong protective effect was observed for long-term oral contraceptive use (HR 0.13, 95% CI 0.02–0.92, p = 0.041).

In the multivariable Cox regression models, levonorgestrel-releasing intrauterine device use was not associated with breast or ovarian cancer risk in neither *BRCA1* nor *BRCA2* pathogenic variant carriers (Supplementary Table S2). Among *BRCA1* carriers, levonorgestrel-releasing intrauterine device use did not significantly affect the risk of breast cancer (HR 0.88, 95% CI 0.65–1.19, p = 0.4) nor ovarian cancer (HR 0.94, 95% CI 0.57–1.54, p = 0.8). Similarly, among *BRCA2* carriers, no significant association was observed between levonorgestrel-releasing intrauterine device use and breast cancer risk (HR 0.97, 95% CI 0.68–1.37, p = 0.8) nor ovarian cancer risk (HR 1.06, 95% CI 0.61–1.84, p = 0.8).

## Discussion

### Summary of the main results

In this large cohort of *BRCA1* and *BRCA2* pathogenic variant carriers, oral contraceptive use demonstrated differential associations with cancer risk by gene type and duration of exposure. Among *BRCA2* carriers, oral contraceptive use was associated with a significant reduction in both breast and ovarian cancer risk, with long-term use conferring the strongest protective effect on ovarian cancer. Among *BRCA1* carriers, use of oral contraceptives did not significantly influence breast cancer risk and showed an insignificant trend toward reduced ovarian cancer risk. Levonorgestrel-releasing intrauterine device use did not affect the risk of either breast or ovarian cancer in *BRCA1* or *BRCA2* pathogenic variant carriers.

### Results in the context of published literature

The protective effect of long-term use of oral contraceptives on ovarian cancer risk in *BRCA1/2* carriers is well established and aligns with prior literature supporting oral contraceptives as an ovarian cancer risk-reducing option in this high-risk population [23]. Oral contraceptives are the most effective non-surgical prevention strategy for high-risk populations, having an inverse association with the incidence of ovarian cancer [7, 24, 25] and resulting in a concomitant decrease in mortality rate and a lifetime reduction in ovarian cancer of approximately 54% [8, 10, 26, 27]. These findings are consistent with the results of the present study.

Notably, no such protective effect was observed with levonorgestrel-releasing intrauterine device use. The observed lack of significant protective effect of levonorgestrel-releasing intrauterine device use on ovarian cancer risk may be explained by its limited systemic hormonal activity and mechanism of action. Unlike oral contraceptive pills, which suppress ovulation through systemic inhibition of the hypothalamic-pituitary-ovarian axis [28], hormonal intrauterine devices primarily act locally within the uterus by releasing levonorgestrel [29]. This results in endometrial suppression, thickening of cervical mucus, and prevention of sperm motility and fertilization, but does not consistently inhibit ovulation, a suspected key process in ovarian cancer evolution [30]. Since one of the leading hypotheses for the protective effect of oral contraceptives against ovarian cancer is the reduction in lifetime ovulatory cycles, which in turn lowers the associated inflammation and epithelial cell turnover in the ovaries [10], the lack of ovulation suppression with intrauterine device use may account for its limited effect in reducing ovarian cancer risk among *BRCA1/2* pathogenic variant carriers.

The relationship between use of oral contraceptives and breast cancer risk among *BRCA1* and *BRCA2* carriers remains complex and subject to ongoing debate. Evidence suggests that the impact of oral contraceptives may be influenced by several factors, including the specific pathogenic variant (*BRCA1* vs. *BRCA2*), age at initiation, duration of use, and timing relative to first full-term pregnancy [9, 21, 23, 31, 32]. In the current study we did not detect a significant association in *BRCA1* carriers, however, we observed an apparent protective association between use of oral contraceptives and breast cancer incidence specifically in *BRCA2* carriers. Nevertheless, this finding should be interpreted with caution, as the duration-specific subgroup analyses did not demonstrate a consistent pattern of association. In particular, no protective effect was observed among long-term users, and the absence of a clear dose–response relationship limits the biological interpretation of this observation. Further large prospective studies are needed to better clarify the relationship between oral contraceptive exposure and breast cancer risk among BRCA2 pathogenic variant carriers. Our findings align with existing evidence suggesting that progesterone signaling has differential effects based on *BRCA* type. Progesterone appears to play a more significant role in breast cancer risk, and its oncogenic impact is better established and more mechanistically understood among *BRCA1* carriers than in *BRCA2* carriers [33–35].

*BRCA1* normally suppresses progesterone receptor activity, and its loss leads to increased progesterone receptor driven proliferation and tumorigenesis. Preclinical studies support the use of progesterone receptor antagonists to reduce this risk in *BRCA1*-deficient models. In contrast, *BRCA2*-related breast cancers are more often hormone receptor–positive, but progesterone’s role in their pathogenesis is less well defined.

The association between levonorgestrel-releasing intrauterine device use and breast cancer risk in *BRCA1/2* carriers also remains largely unexplored, as most existing studies have focused on the general population. A recent systematic review and meta-analysis reported a modest but statistically significant increase in breast cancer risk among levonorgestrel-releasing intrauterine device users in the general population (OR 1.16; 95% CI, 1.06–1.28), with a more pronounced effect observed in women aged 50 and older (OR 1.52; 95% CI, 1.34–1.72) [36]. Similarly, a large national cohort study found a 13% higher risk of breast cancer among levonorgestrel-releasing intrauterine device users compared to non-users (adjusted HR 1.13; 95% CI, 1.10–1.17) [15].

Our study, focusing on *BRCA1/2* pathogenic variant carriers, found no significant increase in breast cancer risk among levonorgestrel-releasing intrauterine device users compared to no hormonal contraceptive. Given the small number of intrauterine device users in our cohort, this result should be interpreted with caution and warrants further investigation in larger, prospective studies.

Finally, it is important to distinguish between hormonal and non-hormonal intrauterine devices when interpreting these findings. In the present study, we evaluated only the levonorgestrel-releasing intrauterine device. Copper intrauterine devices act through a different mechanism, inducing a local inflammatory response within the uterine cavity without systemic hormonal exposure [15]. Consequently, a biological association between copper intrauterine device use and breast cancer risk would be less plausible than with progestin-releasing devices [37]. Indeed, copper intrauterine devices are often recommended as a contraceptive option for women with a history of breast cancer because they avoid hormonal exposure [38]. In contrast to oral contraceptives, intrauterine devices—whether hormonal or non-hormonal—do not reliably suppress ovulation, which is considered a key mechanism underlying the protective effect of oral contraceptives on ovarian cancer risk [9]. Nevertheless, the relationship between intrauterine device use and ovarian cancer risk may be more complex, as proposed mechanisms such as foreign-body inflammatory responses, alterations in the intrauterine environment, and anti-estrogenic effects have also been suggested [39]. Further studies evaluating both hormonal and non-hormonal intrauterine devices in BRCA1/2 pathogenic variant carriers are warranted to better clarify potential differences in cancer risk according to device type.

### Strengths and weaknesses

Our study has several strengths. It utilizes a large dataset from a national healthcare provider and includes detailed clinical and demographic information, allowing adjustment for key confounders. The design enabled stratification by short-, middle- and long-term contraceptive use, according to type of *BRCA1/2* pathogenic variant, thus providing a clearer picture of individual contraceptive effects. Additionally, contraceptive exposures were modeled as time-dependent covariates, which minimizes the risk of immortal time bias and more accurately reflects changes in exposure status over time.

To the best of our knowledge this is the first study comparing not only ovarian cancer risk, but also breast cancer risk, in women with *BRCA1/2* pathogenic variants, according to hormonal contraceptive use. However, several limitations should be considered. First, its retrospective design introduces the potential for selection and information bias. Data were extracted from a large multicenter registry, and certain clinical variables—such as reasons for contraceptive choice, adherence to contraceptive use, and presence of contraindications—were not uniformly available. Additionally, detailed reproductive histories and family history of cancer were not accessible for all participants, limiting our ability to adjust for all potential confounders. Matching was performed according to birth year only and not according to BRCA pathogenic variant type in order to preserve cohort size and statistical power. Although analyses were stratified according to BRCA1 and BRCA2 status, residual confounding cannot be excluded. The relatively small number of levonorgestrel-releasing intrauterine device users may have resulted in imprecise effect estimates and type 2 error cannot be ruled out. Hormone replacement therapy exposure was not modeled as a time-dependent covariate. As a result, the observed protective association of contraceptives for *BRCA2* carriers may be influenced by immortal time bias and should therefore be interpreted with caution.

In addition, multiple outcome and subgroup analyses were performed without formal adjustment for multiple comparisons; therefore, findings with borderline statistical significance should be interpreted cautiously.

### Implications for practice and future research

This study demonstrates that hormonal contraceptive–related cancer risk modification in *BRCA1/2* carriers is gene-specific. Oral contraceptives were protective for breast and ovarian cancer among *BRCA2* carriers, while the lack of benefit with levonorgestrel intrauterine devices emphasizes nonequivalence across methods and the need for further prospective studies.

## Conclusions

Our findings support a protective association between use of oral contraceptives and breast and ovarian cancer among *BRCA2* carriers. While levonorgestrel-releasing intrauterine device use was not significantly associated with reduced ovarian cancer risk or increased breast cancer risk, given the growing use of intrauterine devices as a contraceptive method, further prospective studies with larger cohorts are needed to validate these observations and better define their role in cancer risk reduction strategies for *BRCA1/2* carriers. Our results also suggest that the magnitude and direction of risk modification by hormonal contraceptives may depend on *BRCA* pathogenic variant type and highlight the importance of considering genetic background when evaluating prevention strategies.

## Supplementary Information

Below is the link to the electronic supplementary material.

Supplementary file1 (DOCX 16 KB)

Supplementary file2 (DOCX 14 KB)

## Data Availability

Data available on request from the authors.
