# Supplementary material for: Hormonal contraceptives and cancer risk in women with a BRCA1 or BRCA2 pathogenic variant
Source: Breast Cancer. 2026 Aug 18;33(6):1271–9. doi: 10.1007/s12282-026-01890-z (PMC13695616; doi:10.1007/s12282-026-01890-z)
Supplement: Supplementary file 1 [file 12282_2026_1890_MOESM1_ESM.docx]

# **Supplemantary Table S1: Characteristics of Women who used IUD**

| **Characteristic** | **LNG-IUD users**  **N=511** | **Non LNG-IUD users**  **N=1667** | **P value** |
| --- | --- | --- | --- |
| Mutation type, n (%) | BRCA1= 276 (54%), BRCA2 = 235 (46%) | BRCA1= 990 (59%), BRCA2 = 687 (41%) | 0.024 |
| Smoking status, n (%) | Smoker= 48 (9.4%), past smoker=86 (17%), non-smoker= 377 (74%) | Smoker= 154 (9.2%), past smoker=216 (13%), non-smoker= 1307 (78%) | 0.3 |
| Age at first IUD use, median (IQR) | 37 (32,41) | - | - |
| Year of birth, median (range) | 1,979 (1,940,2,004) | 1,974 (1,938 ,2,005) | <0.001 |
| BMI, mean (SD) | 25.73 (5.16) | 26.06 (5.55) | <0.001 |
| Parity, mean (SD) | 3.39 (1.71) | 3.19 (1.62) | 0.5 |
| HRT use, n (%) | 175 (34%) | 327 (17%) | <0.001 |
| Age at BRCA diagnosis, median (range) | 41 (18,80) | 44 (18,76) | <0.001 |
| Year at BRCA diagnosis, median (range) | 2,021 (2,005,2,024) | 2020 (1999, 2024) | <0.001 |
| Ovarian cancer diagnosis, n (%) | 12 (2.3%) | 88 (5.3%) | <0.001 |
| Age at ovarian cancer, median (range) | 45.5 (24,54) | 50 (23,77) | <0.001 |
| Year at ovarian cancer, median (range) | 2,017 (1,999,2,024) | 2017 (1974,2024) | 0.2 |
| Breast cancer diagnosis, n (%) | 119 (23.3%) | 525 (31.1%) | <0.001 |
| Age at breast cancer, median (range) | 43 (20, 70) | 42 (19, 71) | <0.001 |
| Year at breast cancer, median (range) | 2019 (1975,2024) | 2015 (1975, 2024) | <0.001 |
| Bilateral salpingo-oophorectomy, n (%) | 160 (31%) | 553 (33%) | 0.024 |
| Bilateral mastectomy, n (%) | 58 (11%) | 173 (10%) | 0.06 |
