# Supplementary material for: Hormonal contraceptives and cancer risk in women with a BRCA1 or BRCA2 pathogenic variant
Source: Breast Cancer. 2026 Aug 18;33(6):1271–9. doi: 10.1007/s12282-026-01890-z (PMC13695616; doi:10.1007/s12282-026-01890-z)
Supplement: Supplementary file 2 [file 12282_2026_1890_MOESM2_ESM.docx]

# **Supplementary Table S2. Multivariable Cox regression analyses evaluating the association between levonorgestrel-releasing intrauterine device use and cancer risk according to BRCA pathogenic variant type**

| **Outcome** | **Hazard Ratio** | **95% Confidence Interval** | **p-value** |
| --- | --- | --- | --- |
| Breast cancer – BRCA1 | 0.88 | 0.65–1.19 | 0.4 |
| Ovarian cancer – BRCA1 | 0.94 | 0.57–1.54 | 0.8 |
| Breast cancer – BRCA2 | 0.97 | 0.68–1.37 | 0.8 |
| Ovarian cancer – BRCA2 | 1.06 | 0.61–1.84 | 0.8 |

Models were adjusted for hormone replacement therapy exposure.
